# 

**Published:** 1916-01

**Authors:** 


					﻿THE
DENTAL
REGISTER
Edited by DrNELVILLE S. HOFF
ANN ARBOR. MICH.
vol.lxx JANUARY, 1916 no. i
THE SAMUEL A. CROCKER CO., Pub.ishers
OHIO DENTAL AND SURGICAL DEPOT
CONRAD BLDG., 18 & 20 W. Seventh St., CINCINNATI, O.
PRICE, ONE DOLLAR A YEAR
Entered at the Post-Office at Cincinnati, O , as second-class mail matter
				

